# Heparan Sulfate Mimetics in Cancer Therapy: The Challenge to Define Structural Determinants and the Relevance of Targets for Optimal Activity

**DOI:** 10.3390/molecules23112915

**Published:** 2018-11-08

**Authors:** Cinzia Lanzi, Giuliana Cassinelli

**Affiliations:** Molecular Pharmacology Unit, Department of Applied Research and Technological Development, Fondazione IRCCS Istituto Nazionale dei Tumori, 20133 Milan, Italy

**Keywords:** heparin, heparan sulfate proteoglycan, heparan sulfate mimetics, non-anticoagulant heparin derivatives, heparanase, cancer therapy

## Abstract

Beyond anticoagulation, the therapeutic potential of heparin derivatives and heparan sulfate (HS) mimetics (functionally defined HS mimetics) in oncology is related to their ability to bind and modulate the function of a vast array of HS-binding proteins with pivotal roles in cancer growth and progression. The definition of structural/functional determinants and the introduction of chemical modifications enabled heparin derivatives to be identified with greatly reduced or absent anticoagulant activity, but conserved/enhanced anticancer activity. These studies paved the way for the disclosure of structural requirements for the inhibitory effects of HS mimetics on heparanase, selectins, and growth factor receptor signaling, as well as for the limitation of side effects. Actually, HS mimetics affect the tumor biological behavior via a multi-target mechanism of action based on their effects on tumor cells and various components of the tumor microenvironment. Emerging evidence indicates that immunomodulation can participate in the antitumor activity of these agents. Significant ability to enhance the antitumor effects of combination treatments with standard therapies was shown in several tumor models. While the first HS mimetics are undergoing early clinical evaluation, an improved understanding of the molecular contexts favoring the antitumor action in certain malignancies or subgroups is needed to fully exploit their potential.

## 1. Introduction

### 1.1. Heparan Sulfate and Heparin in Physiology and Pharmacology

Heparan sulfate (HS) and heparin are members of a class of glycosaminoglycans (GAGs) characterized by closely related linear polyanionic polysaccharidic structures, but distinct functions in both physiology and disease [1,2,3,4,5,6,7]. They are synthesized as long polysaccharidic chains covalently bound to a serine residue that is part of a GAG attachment sequence in a protein core, forming glycoconjugates known as HS proteoglycans (HSPGs). HS, synthesized by most cells in the body, is a ubiquitous component of cell surface- and extracellular matrix (ECM)-associated HSPGs. By interacting with a plethora of proteins through the HS chains, HSPGs are able to heavily affect multiple biological processes with essential roles in development and homeostasis, as well as in many pathological conditions, including inflammation, neoplastic transformation, and cancer progression [7,8,9,10,11,12,13,14].

In contrast to HS, heparin is exclusively produced by mast cells, where it is stored in intracellular granules specifically linked to the proteoglycan serglycin, which controls the activities of many proteases [15,16,17]. Stimulated mast cells eject their granules in a process termed degranulation, leading to the extracellular release of heparin, an effect that was proposed to act as a homeostatic “braking” mechanism limiting the extent of inflammation. Nonetheless, the specific role of heparin in normal homeostasis is yet to be fully elucidated.

**Figure 1:**
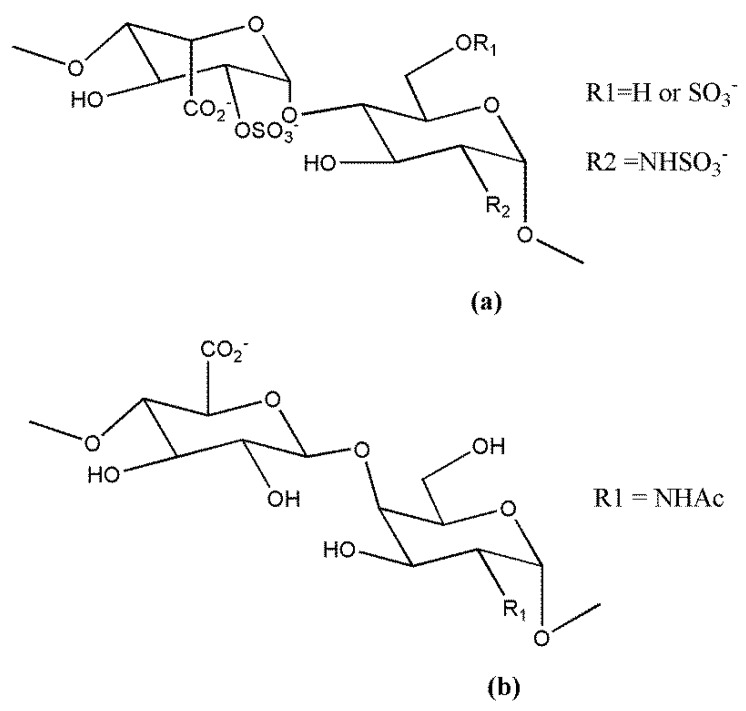
Schematic representation of the major repeating disaccharide (2-*O*-sulfated-l-iduroninyl-1α-4-*O*-*N* sulfated d-glucosamine 1α, also 6-O-sulfated) in heparin (**a**) and (4-*O*-d-glucuronyl-1β-4-*O*-*N* acetyl d-glucosamine 1α) in heparan sulfate (**b**).

HS and heparin display some structural differences between saccharide sequences, as well as sulfation degree and pattern, related to differences between the biosynthetic pathways in mast cells and other cell types. In fact, although the biosynthesis of heparin/HS is a non-template-driven process, it involves at least 22 enzymes and is finely regulated to produce cell- and tissue-specific forms of GAGs [1,2,5,6,7,18,19]. Of note, the sulfatase enzymes, Sulf-1 and Sulf-2, acting extracellularly after transport of HS to the cell surface, trim some of the 6-O-sulfates from HS, whereas they do not modify heparin which it is not a cell-surface GAG [20]. Thus, whereas heparin consists of around 80% of the trisulfated disaccharide containing sulfated iduronic acid (IdoA) and sulfated glucosamine (GlcN), i.e., [(-4) l-IdoA2-*O*-sulfate α(1-4) d-GlcN-sulfate,6-*O*-sulfate α (1-)], the majority of the HS chains are composed of disaccharide repeats containing d-glucuronic acid (GlcA) and d-*N*-acetyl glucosamine (GlcNAc), i.e., [(-4) d-GlcA α (1-4) d-GlcNAc α (1-)], with a lower sulfation degree. Overall, HS structural heterogeneity is greater than that observed in heparin (Figure 1). Although both heparin and HS chain are polydispersed with a broad molecular weight distribution, HS chains are generally longer than those of heparin (~30 kDa vs. ~15 kDa) [4,5,7].

The pharmaceutical formulation of heparin, produced by partial fractionation of natural GAGs derived primarily from porcine intestines or bovine lungs, is used as an anticoagulant for the prevention and treatment of venous thrombosis [7,21]. Experimentally, heparin is widely used as a proxy for HS to dissect the molecular and functional aspects of the HS–protein interactome [22]. Indeed, a large number of proteins, including growth factors, cytokines and chemokines, enzymes and enzyme inhibitors, ECM proteins, and membrane receptors, bind both heparin and HS. Although traditionally classified as heparin-binding proteins, under normal physiological conditions, these proteins actually interact with HS chains of membrane-associated or ECM proteoglycans. Hence, they should be more appropriately termed HS-binding proteins (HSBPs) [23]. The interactions between proteins and HS/heparin are usually dominated by charge–charge interactions between the anionic carboxylate and/or sulfate group of the polysaccharide and basic amino acids of the proteins [22,24], although other interactions also contribute. The frequency, location, and structure of basic amino acids (ariginine, lysine, and histidine) in HSBPs are, consequently, important determinants of their binding properties [25,26]. However, the interaction between HS/heparin and their binding proteins may also be influenced by post-translational modifications, GAG heterogeneity, cationic association, hydrogen bonding, and van der Waals contacts [24,27]. Several studies contributed to highlighting the complexity of HS/heparin–protein interactions. Ori et al. [28] compiled a list of 435 nonredundant human HSBPs in the HS/heparin interactome, which include members of important protein families, such as growth factors, cytokines, and morphogens. Rudd et al. [22] demonstrated, using a bioinformatics survey of 437 HSBPs, that there are no extensive, conserved HS/heparin-binding sequences; rather, there are a number of shorter, more widely spaced sequences that may work in unison to form HS/heparin-binding sites on the protein surface. These also involve non-polar aminoacids and others. The study supports the relevance of the three-dimensional arrangement of these conserved motifs on the protein surface rather than the primary sequence *per se*.

Heparin, as a highly sulfated form of HS, can outcompete HS for binding to protein ligands. Actually, the ability of heparin and derivatives to affect the physiological function of HS underpins their multiple therapeutic applications. Indeed, heparin and related drugs, by virtue of their pleiotropic effects, are being exploited for clinical uses beyond anticoagulation and antithrombotic activities, and are being developed for a wide range of disorders, including cancer [7,12,29,30,31].

### 1.2. Pleiotropic Effects of Heparin and Heparan Sulfate Mimetics in Cancer Therapy

The well-established use of unfractioned heparin and low-molecular-weight heparins (LMWHs) in clinical practice, for the prophylaxis of venous thromboembolism in cancer patients suggested their potential anticancer, antiangiogenic, and antimetastatic activity [32,33,34,35,36,37,38]. In fact, a few retrospective analyses of clinical studies, as well as prospective trials, support a beneficial effect of heparins on cancer patient survival [32,33,34,35,38]. Since hypercoagulability, hemodynamic changes, and endothelial dysfunction, the so-called “Virchow’s triad”, are common cancer features, it is difficult to discriminate and evaluate the contribution of the anticoagulant activity from other biological activities of heparins to patients’ survival [36,39]. Ongoing clinical trials, specifically designed to test the effect of LMWHs in the prevention of metastasis in adjuvant or preoperative settings, are expected to provide informative insights [37].

Anticoagulant activity and side effects (e.g., bleeding and heparin-induced thrombocytemia) limit long-term treatment with heparins in anticancer therapy. Nonetheless, the identification of a low abundance, high-affinity, pentasaccharide within the heparin chain, crucial for antithrombin (AT) binding and activation, opened new therapeutic perspectives [40,41]. These studies, and the clear preclinical evidence of antitumor activity displayed by heparin and several derivatives in cancer models independent of the anticoagulant and antithrombotic properties, prompted investigation of the complex structure–activity relationship of heparin to disclose structural determinants of anticoagulant, antiflammatory, and antitumor/antimetastatic effects [42,43,44,45,46]. An intensive synthetic effort led to the production of heparin/HS derivatives characterized by the ability to mimic heparin non-anticoagulant activities. Evidence for a role of HSPGs in essentially all aspects of tumor biology prompted the development of this class of compounds as anticancer therapeutics.

The beneficial antitumor effects of heparin and HS mimetics are presumed mainly to be related to their direct and/or indirect interference on heparanase activity, adhesion molecules, the tissue factor pathway, and signaling triggered by chemokines/cytokines, growth factors, tissue-degrading enzymes, or endosulfatases. Given the increasingly recognized clinical significance of heparanase, the HS-specific endo-β-d-glucuronidase implicated in multiple aspects of tumor growth and progression [47,48,49,50,51,52], extensive efforts were made to identify agents targeting the HS-degrading activity of this enzyme [53,54,55,56,57]. Accumulating evidence indicates, however, that heparin derivatives and HS mimetics can affect tumor biological behavior by exerting pleiotropic effects through a context-specific mechanism of action based not only on heparanase inhibition, but also on the counteraction of HSPG functions.

## 2. Heparan Sulfate and Cancer

HSPGs are structural and functional components ubiquitously expressed at the cell surface and in the ECM. Nuclear localization was also described in tumor and stromal cells [3,58]. Cell surface-associated HSPGs include four transmembrane syndecans and six glycosylphosphaditylinositol membrane-anchored glypicans. Agrin, perlecan, and collagen XVIII are basement membrane HSPGs, whereas serglycin is found in intracellular secretory vescicles [3,9]. A complex machinery devoted to the synthesis of HSPG core proteins and to the synthesis and post-translational editing of HS polysaccharidic chains, confers these molecules a marked structural diversity and the ability to interact in a specific way with a vast array of HSBPs. The unique structural features of HS, contributed by the coordinated action of several enzymes (e.g., glycosyl- and sulfo-transferases, endosulfatases, deacetylases, epimerase, and the endoglucuronidase heparanase), constitute the main molecular bases of the multitude of biological functions of HSPGs [6,19]. Through binding to stromal components, such as laminin, collagen, and fibronectin, HSPGs participate in the maintenance of ECM integrity. In addition, by sequestering HS-binding growth factors, cytokines, and chemokines, HSPGs protect them from protease degradation, provide extracellular storage, modulate their bioavailability, and allow the formation of ligand gradients. Cell surface-associated HSPGs directly participate in cell signaling by functioning as co-receptors for high-affinity growth factor receptors and integrins [8,12,47,48]. Alterations of HSPG core protein expression and HS chains are common in malignant transformation and progression [8]. A major role in the post-translational modification of HS structure, and consequently, its bioactivity, is assumed by heparanase and the endosulfatases, Sulf-1 and Sulf-2. The functions of these enzyme activities during development, homeostasis, tissue repair, and other physiological processes appear to be hijacked in cancer. In fact, a large body of evidence indicates that the altered expression or activity of either heparanase or endosulfatases determines a profound impact on tumor behavior [13,52,56,59].

Upregulation of heparanase, the mammalian endoglycosidase devoted to HS degradation, is observed in essentially all tumor types examined, including carcinomas, sarcomas, and hematological malignancies where, in several cases, it is associated with an aggressive tumor behavior [49,51,54]. Through its enzymatic activity, heparanase contributes to the structural remodeling of the ECM, thus favoring angiogenesis, inflammation, and metastasis, all processes involving cell motility [47,48,49,55,60]. Heparanase cleaves HS at low sulfation sites producing discrete biologically active fragments still able to link HSBPs. This enables growth factor access to receptors and the activation of cell signaling in tumor and stromal cells. Indeed, reported findings suggest that heparanase and HSPGs synergize to foster tumor growth and progression. This aspect was clearly described for the heparanase/syndecan-1 axis which regulates HSPG expression, clustering, shedding, and growth factor signaling. Through this co-operation, heparanase and syndecan-1 control tumor cell growth, adhesion, spread, and signaling at a distance through exosomes [13,48,60,61,62]. Heparanase has long been known as an extracellularly acting degrading enzyme. However, intracellular functions, mainly associated with nuclear and lysosomal localization, are increasingly reported as influencing heparanase protumorigenic activities [63,64,65,66]. Moreover, evidence is mounting that heparanase accomplishes part of its pleiotropic effects through HS- and enzymatic activity-independent mechanisms [60].

Similarly to heparanase, the two extracellular endosulfatases, Sulf-1 and Sulf-2, were found to be dysregulated in a wide range of human malignancies where they affect the tumor microenvironment and cell signaling by modifying the structure and function of HS. Both sulfatases act by removing 6-O-sulfates from GlcN in the HSPG polysaccaridic chains, thereby influencing their ligand-binding capacity. They are unique among the sulfatase enzyme family in that they are endo-sulfatases and act extracellularly [59,67]. Despite a similar HS selective catalysis, the two enzymes regulate cell signaling mediated by HS-binding growth factors in opposite ways in several tumor types. Indeed, Sulf-1 and Sulf-2 have been reported widely as tumor suppressors and tumor promoters respectively, although conflicting data in a few studies challenged this concept [59].

The role of HS and its structural modifications in cancer has been examined extensively in several excellent reviews [8,9,10,11,12,13,14]. In the following sections, we describe a few examples of cancer-related processes critically regulated by HS and/or heparanase and potential targets of heparin/HS mimetic-based therapies.

### 2.1. Inflammation

The existence of a connection between inflammation and cancer has long been recognized. Not only do chronic inflammatory states predispose one to the development of some tumors, but tumors that initially occur in non-inflammatory contexts even present inflammation features fueled by malignant cells. In turn, inflammatory components of the tumor microenvironment, including infiltrating immune cells, supply bioactive molecules able to foster early stages of tumor growth, as well as progression, favoring angiogenesis, tumor cell invasion, and distant dissemination [68]. It is now known that key events in the inflammatory process, including leukocyte recruitment, adhesion, rolling on the endothelial cell surface, and transmigration into inflamed tissue, are dependent on the interaction of HS with inflammatory modulators (i.e., chemokines, selectins, and integrins) and regulated by the HS-specific enzymatic activity of heparanase. A role for HS and heparanase in inflammation is further supported by the anti-inflammatory effects of HS mimetics observed either in preclinical or clinical settings [11,50,69,70,71].

In the initial phase of inflammation, the interaction between HS present on the endothelial cell surface and L-selectin, an adhesion molecule expressed by leukocytes, is an essential step allowing recruitment and rolling of leukocytes on the inflamed endothelium [11,72]. Both recruitment and infiltration of inflammatory cells through the vessel walls are regulated by the presentation of chemokines on the endothelial cell surface by HSPGs. The specificity and fine-tuning of these processes are modulated by the high level of structural diversity of HS chains, as well as by the vast array of inflammatory HS-binding chemokines [11,73]. Confirming the essential role of endothelial HS in these phases of inflammation, in vivo loss of functional HS due to inactivation of the biosynthetic enzyme N-deacetylase and N-sulfotransferase-1 was shown to result in reduced l-selectin binding to endothelial cells, decreased chemokine binding, and transcytosis, as well as decreased neutrophil infiltration upon different inflammatory stimuli [72]. Moreover, as demonstrated by Massena et al., HS also provides directionality to the intraluminal crawling of immune cells toward the site of transmigration by forming a chemotactic gradient sequestered on endothelial cells [74]. Following the leukocyte transendothelial migration, also assisted by the apical and basolateral HSPG-mediated chemokine gradient and HS interactions with selectins and integrins [75], the subendothelial basement membrane represents an additional major obstacle to further migration. In this phase, the enzymatic activity of heparanase, locally expressed by different cell types (immunocytes, as well as endothelial and epithelial cells), plays an important role facilitating leukocyte extravasation through the basement membrane by degrading HS [76]. Moreover, an indirect effect enhancing the degradative process could be further provided by nuclear heparanase through its effect on the HSPG-mediated regulation of gene transcription with the induction of matrix metalloproteinase expression [64].

The role of heparanase in inflammation is complex and still not completely understood [76,77]. Beyond its HS-degrading function facilitating leukocyte migration through the basement membrane and ECM, heparanase was implicated in macrophage activation via modulation of the Toll-like receptor (TLR) signaling pathway, resulting in inflammatory cytokine induction and release. Heparanase-dependent TLR stimulation was suggested to be mediated by the enzymatic production of HS fragments, although non-enzymatic mechanisms were also brought into play [78,79]. Since, distinct from HS fragments, intact HS inhibits TLR4 and macrophage activation, it was suggested that degradation of the ECM and cell surface HS by heparanase also contributes to macrophage activation by relieving constraints on TLR4 function [80,81]. The key role of heparanase in inflammation-associated cancers is supported by its induction in several tumor-predisposing inflammatory disorders before the occurrence of malignancy [50,76]. Studies performed in mouse models of colon and pancreatic carcinomas exemplified how heparanase may represent a mechanistic link coupling inflammation and cancer [81,82]. Lerner and collaborators [81] showed that, in a mouse model of ulcerative colitis-associated colon cancer, heparanase drives a vicious cycle powering chronic colitis and tumorigenesis. Produced by the inflamed colon epithelium, heparanase activates macrophages which, in turn, contribute to heparanase induction and activation by secreting tumor necrosis factor alpha (TNF-α) and cathepsin L. This self-sustaining cross-talk, thus, prevents inflammation resolution, creating a tumor-promoting microenvironment. A chronic state of aseptic inflammation is implicated in pancreatic ductal adenocarcinoma (PDAC), a tumor in which heparanase overexpression was linked with increased aggressiveness [83,84]. Using a mouse model of PDAC, Hermano et al. [82] found that heparanase overexpression is associated with the infiltration of macrophages characterized by a protumor phenotype, as indicated by the enhanced production of cytokines relevant in PDAC pathogenesis. The observation that human PDAC specimens overexpressing heparanase also show high levels of macrophage infiltration supports the clinical relevance of these findings and suggests a role for the enzyme in guiding the tumor-promoting action of tumor-associated macrophages.

### 2.2. Proliferation, Angiogenesis, and Metastasis

In the pathological conditions of tumorigenesis and tumor progression, where cell mechanisms governing the physiological controls are lost as a result of gene alteration, the tumor stroma, composed of cancer-associated fibroblasts, inflammatory and endothelial cells, pericytes, and the ECM, plays an important role providing a permissive environment [85]. HSPGs, as functional components of the cell surface and ECM endowed with high capacity to bind growth factors, cytokines, chemokines, enzymes, and adhesion molecules, represent “hub molecules” able to modulate key tumor processes such as proliferation, invasion, angiogenesis, and distant colonization. Roles in specific cancers were described for individual HSPGs [8,9,12,54]. HS-binding growth factors regulate multiple carcinogenesis processes being either mitogenic or pro-angiogenic (e.g., platelet-derived growth factor (PDGF), fibroblast growth factor (FGF), and vascular endothelial growth factor (VEGF)) and promoting tumor cell survival, invasiveness, and metastasis (e.g., hepatocyte growth factor (HGF), epidermal growth factor (EGF), and insulin-like growth factor (IGF)) [10]. As previously mentioned, heparanase increases the bioavailability of HS-bound growth factors via its HS-specific degrading activity. Studies performed in a heparanase transgenic mouse model correlated the overexpression of heparanase with the upregulation of HS sulfation, which enhanced the interaction with FGFs and their receptors, thus suggesting a further mechanism whereby heparanase can promote growth factor action in tumors [86]. Notably, heparanase functions independent of its enzymatic activity were implicated in the upregulation of tissue factor and growth factors (i.e., VEGFC, VEGFA, and HGF) [87,88,89] and in the activation of signaling molecules such as EGF receptor (EGFR) and Src [90]. Heparanase can promote an aggressive tumor behavior also through an indirect mechanism by enhancing HSPG ectodomain proteolytic shedding, which converts a membrane proteoglycan co-receptor into a soluble paracrine bioactive effector able to potentiate the activity of HS-binding growth factors (e.g., VEGF and HGF) [91]. Interestingly, recent insights into the effects of heparanase-induced shedding of syndecan-1, an HSPG implicated in the pathobiology of multiple myeloma, revealed a unifying mechanism whereby heparanase expression promotes different aspects of disease progression [92]. It was previously shown that syndecan-1 proteolytic shedding by matrix metalloproteinase 9 (MMP-9) is favored by the heparanase-mediated trimming of HS chains and that heparanase upregulates MMP-9 expression [93,94]. Jung and collaborators demonstrated that a syndecan-1 juxtamembrane site, exposed upon shedding in endothelial and myeloma cells, binds both VEGF receptor 2 (VEGFR2) and very late antigen 4 (VLA-4). Coupling the receptor tyrosine kinase to the integrin activates cell signaling and triggers an invasive phenotype, thus promoting both angiogenesis and metastasis [77,92].

An additional form of cooperation between heparanase and syndecan-1 in the regulation of exosome biogenesis was first reported by the group of Sanderson in a study performed using multiple myeloma cell models with high and low heparanase expression [95]. Further mechanistic insights in breast cancer cells, reported in a study by Roucourt et al. [96], demonstrated that, through its HS-degrading activity, heparanase favors syndecan-1 clustering and formation of a complex containing syndecan-1, the scaffolding protein syntenin, and α-1,3-mannosyltransferase (ALG-2)-interacting protein X (ALIX), a component of the endosomal-sorting complex required for transport (ESCRT) which drives exosome production [97]. Another membrane-associated HSPG, glypican-1, was found specifically enriched in circulating cancer cell-derived exosomes and was identified as a potential biomarker to detect early stage pancreatic cancer [98]. The involvement of heparanase and HSPG in the production, composition, and docking of exosomes from tumor and stromal cells, eventually triggering an aggressive phenotype [95], expands the known impact of heparanase/HSPGs axes in tumor biology and further supports their roles as therapeutic targets. Accumulating evidence suggests that exosomes, defined as “extracellular signalosomes” [99], by delivering their cargo of proteins and nucleic acids to recipient cells in the tumor stroma or at distant organs, act as key players in tumor progression. In fact, they were implicated in epithelial–mesenchymal transition (EMT) signaling, pre-metastatic niche formation, tumor immune escape, and drug resistance (reviewed in References [99,100,101]).

Translocation to the nucleus in tumor and stromal cells is another mechanism whereby cell-surface HSPGs either in their full-length or shed forms can regulate cell functions and tumor–host cross-talk [58,102]. Through this cellular trafficking, syndecan-1 participates in the nuclear delivery of growth factors (e.g., HGF and FGF) and heparanase [103]. Moreover, cooperating with these molecules, HSPG can modulate gene expression by acting at different levels, e.g., the chromatin structure by inhibiting histone acetylation and histone acetylase (i.e., p300) activity [64,102]; or the transcription machinery by interfering with the binding of transcription factors to DNA or by blocking DNA topoisomerase I [104,105]. Although the presence of HS, HSPGs, and heparanase in the cellular nuclear compartment has been known for several years, their functional significance is still incompletely understood. Actually, effects of HSPGs appear to be tissue- and tumor type-dependent. Recently, mutated syndecan-1 unable to translocate to the nucleus was used to address functions and molecular pathways related to nuclear localization in fibrosarcoma cells. By combining transcriptomic and proteomic approaches with functional assays, Szatmari and colleagues identified *EGR1*, *NEK11*, and *DOCK8* as genes responsive to syndecan-1 nuclear translocation and the TGF-β1 pathway as playing a role in the inhibition of cell proliferation and cell-cycle progression [106].

Heparanase was reported to influence gene expression through various direct or indirect mechanisms. Through an enzyme-independent signaling function, mediated by the C-terminal domain, heparanase enhances the activation of protein kinases, ultimately increasing the transcription of genes associated with tumor progression, such as *VEGF-A* and *VEGF-C* [60]. In multiple myeloma models, heparanase was found to represent a critical factor regulating levels of HS and syndecan-1 in the nucleus. Loss of nuclear syndecan-1 was shown to be induced by elevated levels of heparanase, which resulted in a significant increase in histone acetylase activity, leading, in turn, to stimulation of the transcription of genes (i.e., *VEGF* and *MMP-9*) that drive aggressive tumor behavior [64,107]. These findings are in agreement with the observation that HS can negatively regulate histone acetylase activity [108] and support nuclear syndecan-1 as a transcriptional repressor.

In addition to histone acetylation, nuclear heparanase was reported to be able to modulate histone H3 methylation, another key epigenetic process regulating gene expression. Using human Jurkat T cells as a model system to study the transcriptional regulation of immune response genes, He and collaborators [65] found evidence that heparanase associated with chromatin may form an active complex with RNA polymerase II and histone lysine-specific demethylase-1 modifying the H3 methylation pattern. Notably, gain- and loss-of-function studies showed that heparanase is essential for the transcription of inducible immune response genes, including genes required for T-lymphocyte effector function (cluster of differentiation 69 (*CD69*), interleukin 2 (*IL-2*), and interferon gamma (*IFNγ*)) and migration (*MMP-2* and *MMP-14*). It remains to be established whether this mechanism is similarly active in tumor cells.

Mechanistic details and the functional role of the nuclear translocation of heparanase remains incompletely understood. Heparanase induction of HGF and C–X–C motif chemokine ligand 10 (CXCL10) in multiple myeloma appears not to rely on the endo-β-d-glucuronidase enzymatic activity [89,109]. Moreover, counterintuitively to the generally accepted notion of a pro-malignant action of heparanase, nuclear localization was correlated with differentiation in various tumor cellular models [110] and with a favorable outcome in patients with head and neck cancer [111]. Based on in vitro and in vivo studies with melanoma models, Yang and colleagues proposed a model whereby heparanase could exert a dual function with a protumorigenic and a tumor-suppressive activity in the extracellular and the nuclear compartment, respectively [66].

### 2.3. Therapeutic Resistance

Several lines of evidence indicate that tumor sensitivity to drug treatment can be affected by altered expression of cell-surface HSPGs and/or heparanase [51,56,63]. Mechanisms underlying this effect are still not well understood and are likely context-dependent. In gastric carcinoma cells, glypican-3 overexpression was implicated in the decreased drug accumulation associated with atypical multidrug resistance [112]. On the other hand, high glypican-1 expression was found to be related to chemoresistance in patients with esophageal squamous cell carcinoma, and further in vitro studies with cellular models of this tumor suggested a specific role for the HSPG in cisplatin resistance without alteration of drug availability [113].

In breast cancer and multiple myeloma, syndecan-1 overexpression correlates with aggressive phenotype and poor prognosis and was implicated in resistance to either cytotoxic or targeted therapeutics [61,62,114,115,116,117]. Gotte and collaborators [118] noted a relationship between intensity of syndecan-1 immunostaining in pre-chemotherapy breast cancer biopsies and decreased response to treatment with cyclophosphamide and cisplatin. In in vitro studies [119], sensitivity of breast cancer cells to the anti-human ErbB2 (HER2) monoclonal antibody, trastuzumab, was associated with the availability of HS on the cell surface and with the ability of HS to elicit the antibody response by forming a ternary complex with trastuzumab and HER2. In these cellular models, high levels of exogenous heparanase induced resistance to the antibody and the release of HS, suggesting a mechanism whereby HS, capturing trastuzumab in the medium, prevents the formation of the ternary complex. Again, cooperation between syndecan-1 and heparanase, through indirect induction of HSPG shedding, and consequent enhancement of HS-binding growth factor signaling, was suggested to play a role in resistance of colorectal cancer cells to several conventional chemotherapeutics [120]. An additional report implicated high levels of heparanase in resistance of metastatic breast cancer models to the small-molecule tyrosine kinase inhibitor of HER2 and EGFR, lapatinib, through the activation of a compensatory EGFR-dependent pathway [121].

Further studies suggested a role for heparanase overexpression in tumor protection against radiotherapy. In pancreatic cancer models, ionizing radiation was shown to upregulate heparanase by downregulating the transcription repressor *EGR1*, ultimately leading to the enhanced invasive capability of tumor cells [122]. In cervical cancer models, heparanase expression was shown, by gain- and loss-of function experiments, to enhance angiogenesis and radiation resistance through the hypoxia-inducible factor 1 (HIF1) pathway [123].

Recent evidence implicates intracellular lysosomal heparanase in the modulation of autophagy, a catabolic pathway maintaining homeostasis in normal cells and dysregulated in several tumors, promoting cancer cell survival [63,124]. Vlodavsky and his group showed that heparanase co-localizes with the autophagy marker, lipid-modified microtubule-associated protein 1A/1B-light chain 3 (LC3-II), and that autophagy extent correlates with heparanase expression levels [63,125]. In this study, resistance to stress (i.e., amino-acid starvation) and chemotherapy (i.e., cisplatin) was induced by upregulation of heparanase and was shown to be mediated, at least in part, by increased autophagy.

An additional interesting discovery in recent years is that not only can alterations of the heparanase/HSPG system in tumors contribute to modulating response to drug treatments, but chemotherapy, in turn, can promote the heparanase/HSPG axis functions. Clinical anti-myeloma drugs were shown to induce syndecan-1 shedding in multiple myeloma cell cultures and in tumor xenografted mice, as well as the upregulation of heparanase expression and its release into conditioned medium. Soluble heparanase, in turn, could be taken up by both tumor cells and macrophages, enhancing the expression of pro-tumorigenic genes. Clearly, these chemotherapy side effects have the potential to promote re-establishment of a microenvironment permissive for tumor relapse. Indeed, gene expression analyses of myeloma cells from patients subjected to sequential rounds of chemotherapy revealed that tumor cells that survived expressed elevated levels of heparanase. Moreover, the forced high expression of heparanase in myeloma cells induced chemoresistance [126,127]. Alishekwits et al. added an additional element to the emerging connection among chemotherapy, heparanase/HSPGs, inflammation, and tumor resistance to therapy, re-growth, and metastasis [128]. Using breast cancer models, they demonstrated that paclitaxel treatment induced an increase in VEGF-C levels, which stimulated tumor-infiltrating macrophages to release cathepsin. They proposed that the resulting proteolytic activation of heparanase further promoted the expression of VEGF-C, ultimately leading to VEGFR3-dependent lymphangiogenesis and metastasis.

## 3. Non-Anticoagulant Heparin Derivatives and Oligosaccharidic HS Mimetics

The molecular basis of the anticoagulant action of heparin lies in its ability to bind to and enhance the inhibitory activity of the serpin antithrombin (AT) against several serine proteases of the coagulation cascade, most importantly factors IIa (thrombin), Xa, and IXa [21]. Moreover, heparin can act through other serine protease inhibitors such as heparin cofactor II, protein C inhibitor, and tissue factor pathway inhibitor, representing other endogenous anticoagulant proteins.

A simplistic view of the interaction between heparin and AT involved a specific high-affinity pentasaccharide sequence (ATBR), present in only one third of the unfractioned heparin chains, containing the typical 3-O-sulfated GlcN and, optionally, the 6-O-sulfated GlcNSO_3_3,6SO_3_ residue [18,21]. Further studies suggested the extension role of the active pentasaccharide sequence on AT-binding properties of heparin oligosaccharides and the generation, by depolymerization processes, of many structural variants influencing the AT-binding properties and regulating the interaction with several other proteins [129]. Heparin species with decreased or negligible anticoagulant properties can be obtained either by removing chains with high affinity for the AT-binding sequence or by inactivating critical functional groups or units of the ATBR through depolymerization or other chemical modification, such as desulfation or glycol-splitting [18,130,131]. Therefore, a drastic decrease in anticoagulant properties results from removal of the 3-O-sulfate of the central ATBR GlcNSO_3_3,6SO_3_ residue or modifications of the GlcA residue in the pentasaccharidic sequence by reduction of its carboxyl group or by cleavage of the bond between its two vicinal hydroxyl groups. In particular, periodate oxidation/borohydrate reduction of non-sulfated uronic acid residues of heparin represents an effective way of inactivating the ATBR, leading to the so-called glycol-split heparins. Since periodate oxidation of GlcA component of the ATBR sequence hinders the interaction with AT [132], the residual anticoagulant activity may result from non-AT-binding-mediated mechanisms. On the other hand, N- and O-desulfation in heparin sequences located outside the ATBR can also impair thrombin inhibition mediated by heparin cofactor II and the release of vascular tissue factor pathway inhibitor [130].

### 3.1. Non-Anticoagulant Heparin Derivatives

Some breakthrough experimental studies paved the way for the design and development of non-anticoagulant anticancer heparin derivatives and contributed to the identification of the pleiotropic mechanisms underlying their antitumor/antimetastatic effects.

Bar-Ner et al. [133] described the heparanase inhibitory effects of non-anticoagulant heparin species and suggested some structural requirements for endo-β-d-glucuronidase inhibition. Indeed, they indicated that the enzyme inhibition was dependent on polysaccharide size, degree and distribution of sulfate groups, and substitution at the N-positions of hexoseamines.

N-acetylated, N-desulfated heparin, as well as N-resulfated N- and O-desulfated heparin, significantly reduced lung colonization abilities of the highly metastatic B16-BL6 mouse melanoma, and similar effects were described with the O-sulfated, N-desulfated, N-acetyl, or N-hexanoyl heparin derivatives, all endowed with potent heparanase inhibitory activity [134,135,136]. A very LMWH and an LMW fully *N*-succinyl heparin, endowed with low anticoagulant activity, were found highly effective in decreasing the lung colonization of B16-BL6 melanoma and in prolonging mice survival [137].

**Figure 2:**
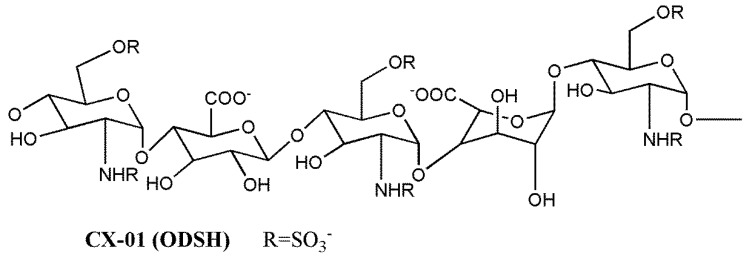
Chemical structure of CX-01 (ODSH) polysaccharide representative of 2,3-*O*-desulfated ATBR.

Lapierre et al. [138] reported the potent heparanase inhibitory activity of 2,3-*O*-desulfated heparin (ODSH/CX-01) (Figure 2) and periodate-oxidized, borohydride-reduced heparin (RO-H), along with their antiangiogenic activity in the chick chorioallantoic membrane bioassay. ODSH exhibited antitumor activity against human Ca-Pan-2 pancreatic adenocarcinoma xenografts and antimetastatic activity in the B16-F10 mouse melanoma model, an effect associated with a significant improvement of mice survival [138,139]. ODSH also inhibited P-selectin-mediated adhesion of human A375 melanoma cells to platelets [140]. Interestingly, the removal of 2-O- and 3-O-sulfate groups, reducing the affinity for AT and increasing the margin of safety, did not substantially harm the anti-inflammatory properties of the agent. ODSH was also able to inhibit complement activation and to interfere with sequential events in leukocyte-mediated inflammation by inhibiting P- and L-selectin, azurocidin, elastase, and G cathepsin [139,141]. The interaction of the vascular adhesion molecule, receptor for advanced glycation end products (RAGE), with its disparate ligands, mediating amplification of inflammatory response, was also disrupted by ODSH. Noteworthy, ODSH infusion in human volunteers produced anti-inflammatory effects without anticoagulation-related adverse effects. Recently, Zheng et al. [142] described a novel mechanism contributing to the ODSH anti-inflammatory properties. The modified heparin inhibits the release from macrophages of the alarmin and nonhistone chromatin-binding protein high-mobility group box 1 (HMGB1), an important inflammatory mediator able to interact with RAGE. ODSH, via direct molecular inhibition of the histone acetyltransferase p300, impairs HMGB1 lysine acetylation and, consequently, its secretion. ODSH (CX-01) is currently in clinical trials, as described in Section 3.3.

Roy and colleagues [143] performed bioactivity screening of a series of size-defined (6–7 kDa) partially desulfated LMWHs generated by regioselective chemical modifications. N-, 6-O-, and 2-O-desulfated derivatives displayed reduced anticoagulant activity and varying affinity toward angiogenic growth factors (i.e., FGF2, VEGF, stromal cell-derived factor 1 alpha (SDF1-α)). The 6-O- and 2-O-desulfated derivatives significantly inhibited murine melanoma B16-F10 colonization to the lung. The N- and 6-O-desulfation decreased the ability to inhibit P-selectin/ligand interaction.

Supersulfated LMWH (ssLMWH) with an average MW of 6300 Da and a sulfate-to-carboxyl ratio of 3.8, obtained via depolymerization concomitant with oversulfation, displayed reduced anticoagulant activity [144,145]. This heparin derivative was shown to interfere with the activity of several pharmacological targets including heparanase, growth factor/receptor axes and proinflammatory molecules (e.g., leucocyte elastase and cathepsin G) [146,147]. In particular, in human synovial sarcoma models, ssLMWH exhibited remarkable in vitro and in vivo antitumor and antimetastatic activities [146]. It dose-dependently inhibited cancer cell colony growth and invasion and downregulated the activation of receptor tyrosine kinases of EGFR, PDGFR, and IGF1R families. Interestingly, the combination of ssLMWH with a small-molecule inhibitor of IGF1R/insulin receptor (IR) produced a synergistic antiproliferative effect, abrogated cell motility, and promoted apoptosis in CME-1 synovial sarcoma cells. In vivo, the drug combination profoundly suppressed CME-1 orthotopic xenograft growth and distant spontaneous lung metastatic dissemination. The latter effect was associated with the presence of aggregates of natural killer (NK) cells in degenerated metastastic deposits and with a trend toward reduced levels of macrophages and neutrophils in lung metastases. These observations suggested the contribution of an immunomodulatory effect to the antimetastatic activity of the ssLMWH combination with the receptor tyrosine kinase inhibitor.

Other supersulfated heparins with low anticoagulant activity (e.g., ssLMWH-19) were reported to strongly inhibit the expression of hepcidin, a key factor in the regulation of iron metabolism also involved in carcinogenesis and metastasis [148].

Interestingly, chemical conjugation of an LMWH with deoxycholic acid led to orally active derivatives which exhibited remarkable anticancer therapeutic potential, thus providing a great advantage in the perspective of chronic treatment regimens [149,150,151]. The presence of deoxycholic acid increased the LMWH hydrophobicity and reduced the flexibility of the molecule, likely causing severe conformational changes of interacting angiogenic factors, thus impairing their biological functions. In particular, oral administration of the LHD4 derivative, characterized by a high deoxycholic acid conjugation ratio and minimal anticoagulant activity, resulted in marked antiangiogenic and antitumor activity in murine SCC7 squamous cell carcinoma and human A549 lung carcinoma models [151].

A periodate-treated, non-anticoagulant heparin carrying a hydrophobic polystyrene chain (NAC-HCPS) displayed antiangiogenic and antimetastatic effects both in vitro and in vivo, reducing lung metastatic colonization from B16 melanoma and Lewis lung cancer (3LL) cells [152]. Moreover, NAC-HCPS significantly inhibited tumor growth and vascularization of subcutaneously implanted 3LL, an effect likely related to its ability to inhibit the endothelial cell growth stimulated by VEGF165, FGF2, or HGF.

Yoshitomi et al. [153] reported the antimetastatic activity of a chemically modified low-anticoagulant heparin (LAC heparin) obtained via the sodium periodate oxidation and sodium borohydride reduction of heparin. By virtue of its reduced anticoagulant activity, it can be repetitively administered via intravenous (i.v.), subcutaneous (s.c). and intraperitoneal (i.p.) injections without severe bleeding complication. The LAC heparin effect on tumor cell dissemination of several murine metastatic models, i.e., 3LL, B16-F10 melanoma, colon 26 carcinoma, and FBJ osteosarcoma, was related to its ability to impair tumor cell adhesion and extravasion in lung capillaries by competitively inhibiting cell-surface HS functions.

**Figure 3:**
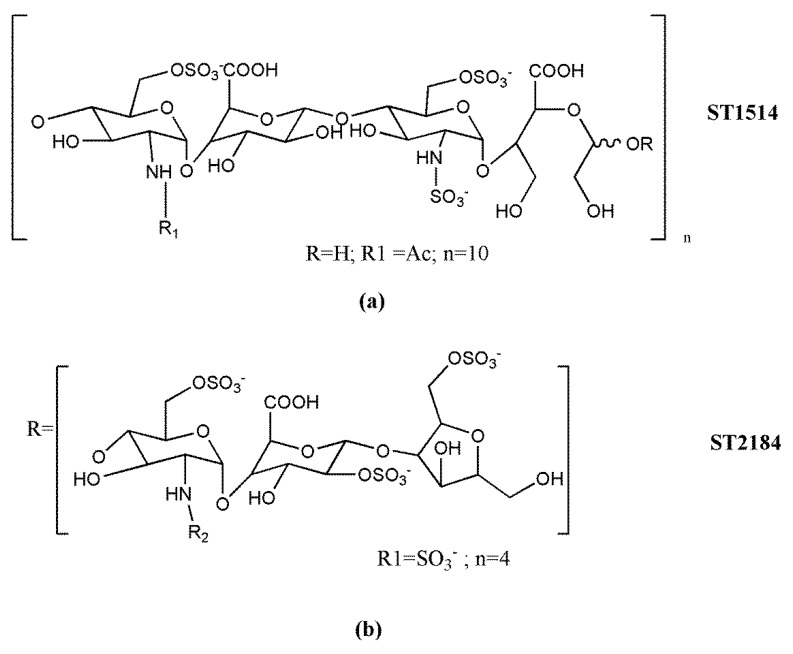
Prevalent structures of the partially (50%) 2-*O*-desulfated glycol-split heparin ST1514 (**a**) and its low-molecular-weight (LMW) derivative ST2184 (**b**).

The non-anticoagulant heparin derivative ST1514 (Figure 3a) was obtained by introducing sulfation gaps along regions of heparin though selective removal of 2-O-sulfate groups to reach a ratio of about 1:1 between sulfated and non-sulfated uronic acid residues. Then, the uronic C(2)–C(3) bonds were split, generating flexible joints along the heparin chains while minimizing cleavage of glycosidic bonds [154,155]. As the splitting reaction also occurs at the level of the essential GlcA residue of the AT binding site, ST1514 was no longer anticoagulant. Moreover, it displayed potent FGF2 antagonist and angiostatic activity. Analogously, ST2184 (Figure 3b), the LMW derivative of ST1514, differing from the precursor mainly by the presence of 2,5 anhydromannitol residues at the chain reducing end, was able to bind VEGF165, exerting marked VEGF165 antagonist activity and antiangiogenic action in vitro [156]. These studies demonstrated that glycol-split heparin chains are more flexible than unmodified ones and conformationally driven to adopt geometries unfavorable for the formation of the ternary complexes with FGF2 or VEGF165 and their receptors [154,155,156]. Both ST1514 and ST2184 inhibited metastatic dissemination to the lung in the B16-BL6 mouse model melanoma, and the LMW derivative significantly reduced angiogenesis of human MeVo melanoma xenografts and potentiated the antitumor activity of a camptothecin derivative [157]. Furthermore, by inhibiting heparanase enzymatic activity, ST1514 significantly reduced wound vascular density and decreased inflammatory response in heparanase-overexpressing transgenic mouse models of wound healing and delayed-type hypersensitivity, respectively [158,159].

**Figure 4:**
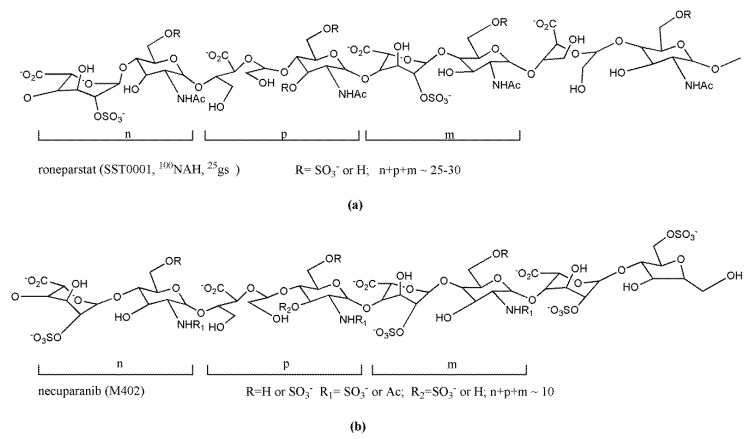
Simplified formulae of representative chains of the glycol-split derivatives roneparstat (**a**) and necuparanib (**b**).

Further chemical modifications performed on glycol-split heparins allowed the identification of derivatives with negligible anticoagulant activity, able to interfere with growth factor-mediated signaling and to efficiently inhibit heparanase. Naggi et al. [160] described the potent heparanase inhibitory activity of glycol-split N-acetyl heparins and, in particular, of the N-desulfated, 100% N-acetylated and 25% glycol-split derivative (^100^NAH, ^25^gs, SST0001, roneparstat) (Figure 4a). Interestingly, these series of derivatives were not susceptible to the cleavage by the endo-β-d-glucuronidase. Moreover, the presence of *N*-acetyl-glucosamine restricted the release of FGF2 from ECM and did not stimulate bFGF mitogenic activity. Further molecular modeling studies showed that glycol-split residues along the heparin chains and mainly located within 6-O-sulfated sequences in the roneparstat molecule act as flexible joints which favor the docking and interaction of these heparin derivatives with multiple HSBPs, including growth factors or heparanase [130,161,162]. In addition, recent studies suggested the existence of multiple heparanase–roneparstat interaction models depending on the drug concentration; whereas a single molecule of roneparstat could interact with the two main heparin-binding domains of heparanase, the drug at high concentration would interact with two heparanase adjacent molecules promoting the enzyme oligomerization [163]. According to the potent heparanase inhibitory activity, roneparstat was shown to efficiently attenuate the lung metastatic colonization of B16-BL6 mouse melanoma cells, which produce both P-selectin ligands and heparanase, whereas it was ineffective on dissemination from the mouse colon carcinoma MC-38 metastatic model which primarily expresses selectin ligands [164]. These findings suggested that heparanase is a primary target of roneparstat in vivo. Roneparstat emerged as a lead compound in this field, demonstrating remarkable antitumor, antiangiogenic, antimetastatic, and immunomodulatory activity in several preclinical models of both hematological and solid tumors [92,117,121,122,165,166,167,168,169,170]. These results prompted its clinical investigation (Section 3.3).

Another heparin derivative rationally designed to have reduced anticoagulant activity is M402 (necuparanib) (Figure 4b) [171], a glycol-split LMWH resulting from nitrous acid depolymerization of heparin followed by periodate oxidation and borohydrate reduction, to give chains bearing glycol-split uronic acid moieties. It exhibits a complex mechanism of action related to its ability to bind with high affinity and to interfere with the biological function of several HSBPs, including P-selectin, VEGF, FGF2, SDF1-α, and heparanase, likely related to the presence of N-sulfated glucosamine. Necuparanib showed promising antitumor/antimetastatic effects in several preclinical models and entered early clinical trials (Section 3.3).

Among the non-anticoagulant glycol-split LMWHs (NAC) obtained from unfractioned heparin by controlled heparinase I depolymerization, NAC8000 and NAC10000 derivatives significantly inhibited BL16-F10 mouse melanoma spreading to the lung [172].

Mousa et al. [173] demonstrated that the non-anticoagulant ultra-LMW heparin (NA-LMWH) obtained by acid hydrolysis of a glycol-split depolymerized heparin preparation, significantly inhibited BL16-F10 mouse melanoma experimental metastases. The increased release of tissue factor pathway inhibitor from vascular endothelial cells induced by NA-LMWH could contribute to its antimetastatic activity. Studies from the same group reported the antimetastatic efficacy of the sulfated low-anticoagulant heparin (S-NACH) in the mouse MPanc96 pancreatic cancer model [174]. S-NACH attenuated cancer cell adhesion to endothelial cells and platelets by inhibiting P-selectin. In addition, in orthotopic pancreatic MPanc96 and SUIT2 models, S-NACH significantly inhibited tumor growth and angiogenesis and enhanced gemcitabine response without any systemic anticoagulant effects. MPanc96 cells exposed to S-NACH showed induction of the antiangiogenic protein thrombospondin-1 and reduction of the X-linked inhibitor of apoptosis protein (XIAP) [175].

Ultra-LMWHs were generated via the physicochemical depolymerization method based on hydrogen-catalyzed radical hydrolysis assisted by ultrasonic waves. They displayed moderate anticoagulant properties related to molecular weight reduction and desulfation induced by this physicochemical method. Moreover, the ultrasonic-assisted radical depolymerization of heparin produces a random hydrolysis of glycosidic bonds preserving antiheparanase activity comparable to that of commercial LMWHs [176].

### 3.2. Oligosaccharidic HS Mimetics

**Figure 5:**
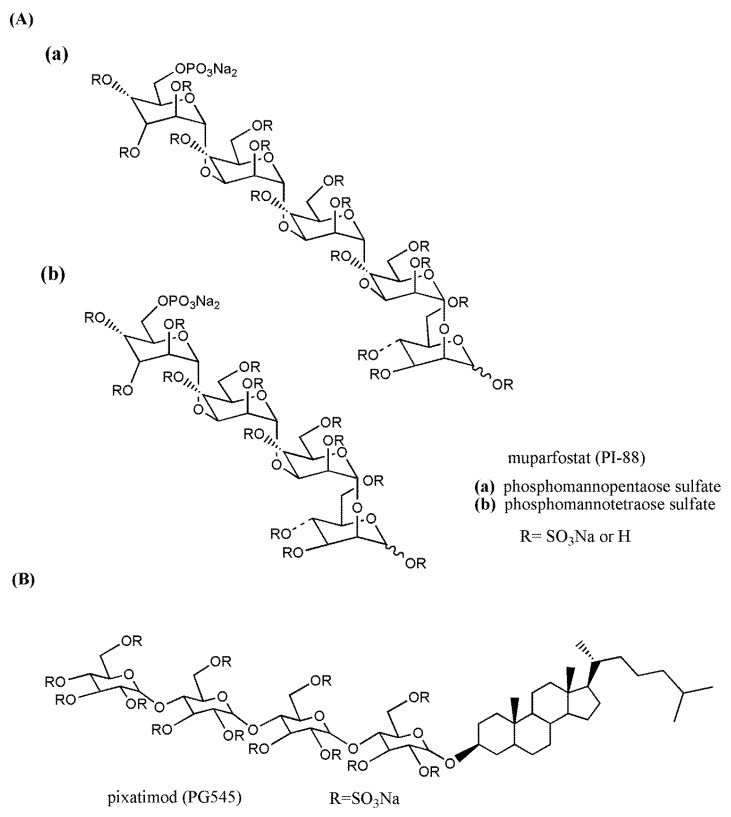
Schematic representation of muparfostat (oligosaccharide mixture) (**A**) and the chemical structure of pixatimod (**B**).

The phospho-sulfo-mannan PI-88 (muparfostat) (Figure 5A) was identified in a comprehensive discovery program among sulfated oligosaccharides endowed with antiangiogenic and antiheparanase activity [177,178]. PI-88 was obtained via sulfation of a phospho-mannan complex produced in cultures of the yeast *Pichia holstii*, as a highly sulfated oligosaccharide mixture, ranging from di- up to hexasaccharides, mostly penta- (60%) and tetrasaccharides (30%). As an HS structural mimetic non-cleavable by heparanase, it inhibited the endo-β-d-glucuronidase activity and prevented the release of proangiogenic growth factors (i.e., FGF1, FGF2, and VEGF) by competing with HS [179]. In addition, displaying high-affinity interactions with several angiogenesis-stimulating growth factors, PI-88 interfered with the ligand–HSPG–receptor ternary complex and, therefore, with signaling activation [180]. It was also shown to induce the release of the endogenous antiangiogenic tissue factor pathway inhibitor [181] and to inhibit the HS 6-O sulfatases, Sulf-1 and Sulf-2 [182]. Although PI-88 maintained anticoagulant activity related to its capacity to enhance heparin cofactor II, it appeared well tolerated in animal studies. Thus, its ability to significantly inhibit tumor growth, angiogenesis, and metastasis in preclinical models prompted its clinical investigation (Section 3.3).

PG545 (pixatimod) (Figure 5B) is a fully sulfated, PI-88-like, tetrasaccharide functionalized with a cholestanyl aglycone, selected as a lead clinical candidate among olisaccharidic HS mimetics of the PG500 series. These compounds provided several advantages over PI-88 being based on anomerically pure, fully sulfated oligosaccharides with a lipophilic aglycone at the reducing end that confers improved biological activity and reduced anticoagulant activity [183]. Indeed, the lipophilic modification in PG545 resulted in improved in vivo pharmacokinetics, allowing less frequent dosing (weekly versus daily) compared with other HS mimetics [184]. PG545 showed antitumor activity in several murine and human solid and hematological tumor models and potent anti-metastatic effect in experimental and spontaneous metastasis models. Of note, beyond its antiheparanase and antiangiogenic activity, the emerging immunomodulatory activity of PG545 represents an additional aspect of the mechanism of action of this HS mimetic [185], which is currently undergoing clinical trials (Section 3.3).

### 3.3. Clinical Candidate HS Mimetics

Based on promising preclinical data, a few heparin derivatives and HS mimetics (hereafter, all functionally referred to as HS mimetics) progressed to clinical evaluation (Table 1).

Vast amounts of the literature describing the biological effects of such compounds in tumor experimental models were revised in several reviews to which the reader can refer [53,55,56,57,131,192,193,194]. Here, some recently emergent peculiar effects characterizing their preclinical and clinical antitumor activity are highlighted.

The 2,3-*O*-desulfated heparin CX-01 (ODSH) (Figure 1) is undergoing clinical evaluation as an adjuvant to chemotherapy. It has low anticoagulant activity but retains most of the anti-inflammatory properties of heparin [139]. The results of a clinical pilot study preceding phases I/II were recently released [186]. CX-01 was combined with the standard therapy, cytarabine and idarubicin, for the treatment of acute myeloid leukemia. The derivative was well tolerated and the combination was associated with a high rate (92%) of morphologic complete remissions. The rapid hematological recovery was also encouraging, an effect that could be related to the binding and neutralization of platelet factor 4, a negative regulator of megakariopoiesis [195]. Interestingly, parallel in vitro studies suggested that CX-01 may interfere with the CXCL12/C–X–C chemokine receptor 4 (CXCR4) axis implicated in the homing of leukemic stem cells in marrow stromal niches via competitive inhibition for the binding of CXCL12 to the marrow HS [186]. These findings require confirmation in large studies, and clinical trials of CX-01 in combination with cytarabine and idarubicin (phase II) or azacitidine (phase I) for treatment of acute myeloid leukemia are ongoing at present.

The N-acetylated glycol-split heparin roneparstat (SST0001) (Figure 4a) completed a phase I clinical trial in advanced relapsed/refractory multiple myeloma patients who exhausted all available therapies for the disease. The study was the first evaluating a HS mimetic/heparanase inhibitor in a hematological malignancy. The results, recently reported, indicated an excellent safety profile and hints of anti-tumor activity, although evidence of efficacy was beyond the objective of the study [187]. Notably, in preclinical studies, disruption of the heparanase/syndecan-1 axis by roneparstat was strongly correlated with the inhibition of multiple processes critical in multiple myeloma development and progression including growth, angiogenesis, dissemination, and bone disease-associated osteolysis, a major cause of morbidity in these patients [92,165,196]. Preclinical evidence of antitumor activity was shown in either hematological malignancies (multiple myeloma and lymphoma) or solid tumors (e.g., melanoma, breast and pancreatic cancer, and sarcomas) with the most remarkable effects noted particularly in combination treatments where roneparstat was found to potentiate the in vivo action of standard therapies, including conventional cytotoxic and targeted drugs [170]. Interestingly, in vitro studies demonstrated the ability of this HS mimetic to strongly counteract receptor tyrosine kinase-mediated signaling and heparanase-induced expression of genes associated with tumor aggressive phenotypes [127,168].

The glycol-split LMWH necuparanib (M402) (Figure 4b) progressed to a phase I/II trial following promising preclinical data demonstrating antitumor/antimetastatic activity in orthotopic and genetically engineered murine models of breast and pancreatic cancer, in addition to a remarkable effect in combination with cytotoxic chemotherapeutics [171,197,198,199]. The clinical trial, conducted in patients with metastatic pancreatic adenocarcinoma, consisted of a two-part study of necuparanib in combination with nab-paclitaxel and gemcitabine. The results of the first part, phase I, of the study indicated a favorable tolerability and signals of activity, including partial responses and stable diseases, with an overall disease-control rate of 63% [188]. Although encouraging efficacy outcomes were found in the early clinical trial, the subsequent part of the study, phase II, was discontinued after interim futility analysis showing an insufficient level of efficacy.

Among the HS-related compounds specifically developed as antitumor agents, the highly sulfated phosphosulfomannan muparfostat (PI-88) (Figure 5A) was the first to enter clinical trials reaching phase III in patients with hepatitis virus-related hepatocellular carcinoma after surgical resection. Despite preliminary activity shown in previous phase II studies, the phase III trial was concluded following the interim analysis indicating that the drug failed to reach the primary disease-free survival endpoint. However, subsequent protocol analyses after completion of one-year treatment revealed a significant prolongation in disease-free time in the microvascular invasion patient subgroup [189]. The presence of microvascular invasion in hepatocellular carcinoma, comprising 40% of the trial population, represents a prognostic factor associated with higher recurrence rates and lower survival. Interestingly, patient stratification using subgroup analysis in an observational follow up study of a phase II trial of PI-88 in an adjuvant setting for hepatocellular carcinoma also indicated the most significant survival advantage for patients at higher risks of recurrence [200]. These findings, thus, support a possible clinical benefit of muparfostat in subgroups of patients with hepatocellular carcinoma. An experimental basis for muparfostat as adjuvant therapy after liver cancer resection was recently provided by Liao and colleagues, who showed that PI-88 blocked the upregulation of heparanase induced in regenerating liver and plasma after partial hepatectomy in mice harboring orthotopic tumors [201].

Muparfostat was generally well tolerated in animal and clinical studies. However, reported toxicities related to residual anticoagulant activity (e.g., injection site hemorrhage, thrombocytopenia, and bleeding events) prompted the synthesis of second-generation PI-88 analogs (P500 series) aimed at obtaining compounds endowed with more favorable pharmacokinetic profiles and toxicology while maintaining the biological activity [183]. Pixatimod (PG545) (Figure 5B), the lead compound in this series, has low anticoagulant properties and has shown remarkable antitumor activity in several preclinical models associated with the inhibition of heparanase and competition with HS for binding to growth factors [184,202,203,204,205]. In models of ovarian and pancreatic cancer, it also enhanced the anticancer activity of standard chemotherapies [206,207]. Recent reports also ascribe to pixatimod direct proapototic effect by interfering with Wnt/β-catenin signaling in pancreatic cancer cells and endothelial reticulum stress response in lymphoma cells [185,207]. A remarkable aspect of the antitumor activity of this compound pertains to the potent immunomodulatory activity which could be critical for its antitumor effect. Investigation into the mechanism underlying immunomodulation in lymphoma models revealed that pixatimod enhanced cytosine–phosphate–guanine (CpG)-mediated TLR9 activation on dendritic cells, leading to increased production of proinflammatory cytokines (e.g., IL-12) and NK-cell activation [208]. In addition, pixatimod inhibited the infiltration of tumor-associated macrophages and myeloid-derived suppressor cells in experimental pancreatic carcinomas [82,209]. Evidence of immune modulation was also provided by pharmacodynamic data obtained in the phase I monotherapy in advanced cancer patients, recently completed [191]. Moreover, using a syngeneic breast cancer model, Hammond and colleagues demonstrated that pixatimod not only modulated innate immune cells but, in combination with an anti-programmed death 1 (PD-1) checkpoint inhibitor antibody, also enhanced T cell infiltration potentiating the antitumor effectiveness [210]. Based on these properties, a phase I trial of pixatimod in combination with the anti-PD-1 inhibitor, nivolumab, in patients with advanced solid tumors and metastatic pancreatic cancer is ongoing. Further bringing HS competition/heparanase inhibition closer to the clinic, in a recent paper, pixatimod was tested in lung cancer patient-derived xenografts (PDX). It was found to be highly effective in reducing tumor growth and lymph node metastases in >85% of cases. It was also effective in PDX that did not respond to cisplatin, suggesting the possibility that HS/heparanase targeting treatment can be applied where conventional cytotoxic chemotherapy fails [211].

## 4. New Molecules and Perspectives

Porcine and bovine tissues are still the sole sources for commercial heparin. Difficulties/hurdles related to sourcing, isolation, and purification of heparin continue to be faced in order to obtain a pharmaceutical-grade drug or intermediates with guaranteed safety and efficacy. To circumvent some manufacturing problems, as well as the risk of contamination with pathogens (e.g., mammalian prionic proteins and viruses) or other dangerous polysaccharides (e.g., oversulfated chondroitin sulfate), intensive efforts were made to identify alternative sources of heparin-like compounds, as well as synthetic derivatives endowed with similar biological activities but devoid of side effects [212,213,214].

### 4.1. HS and Heparin-Like GAGs from Marine and Terrestrial Invertebrates

**Figure 6:**
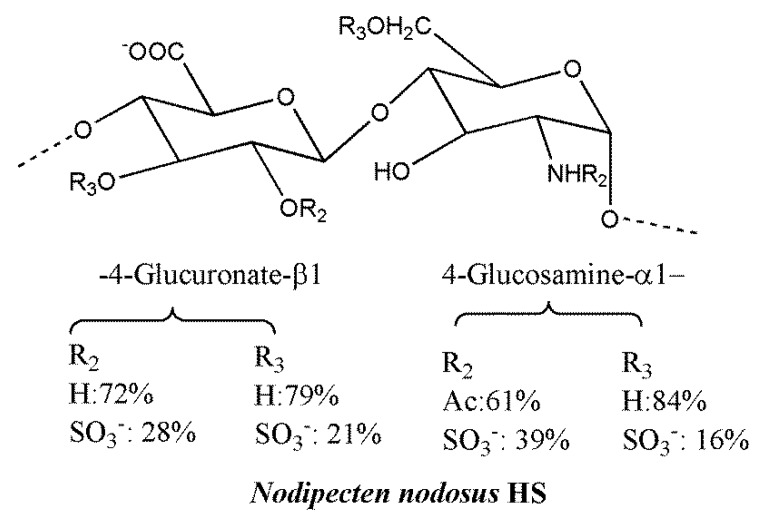
Schematic representation of the major disaccharide unit of *Nodipecten nodosus* heparan sulfate.

HS and heparin-like GAGs from marine prokaryotes and eukaryotes characterized by unique structural chemodiversity offer the opportunity of evaluating biological activities of novel materials in native form or after their selective chemical modification [214]. For instance, heparin-like polymers characterized by low anticoagulant activity and devoid of bleeding effects, but endowed with anti-inflammatory and antimetastatic activities, were identified in mollusks cultivated on a large scale [215]. The unique HS from the bivalve mollusk, *Nodipecten nodosus* (Figure 6), displays P-selectin and heparanase inhibitory properties and does not induce bleeding effects [216,217]. The heterogeneous polysaccharide is characterized by the major disaccharide, 4-*O*-d-GlcA β1-4-d-GlcN 1α, showing partial and random 2- and/or 3-O-sulfation and/or 2-N and 6-O-sulfation, respectively. It drastically attenuates experimental metastases induced by mouse 3LL lung carcinoma cells, an effect associated with the inhibition of platelet–tumor cell complex formation in blood vessels. In addition, the mollusk HS was able to reduce inflammatory cell recruitment suggesting L-selectin inhibitory properties, which may contribute to the reported inhibition of lung metastatic colonization of human colon carcinoma LS180 cells [213].

Acharan sulfate (AS) is a GAG from the giant African snail, *Achatina fulica*, characterized by a primary repeating disaccharide structure α-d-*N*-acetylglucosaminyl-2-*O*-sulfo-α-l-iduronic acid. It exhibited antitumor and antiangiogenic activity in vitro and in vivo, significantly reducing the tumor growth of 3LL lung carcinoma and sarcoma 180 [218,219]. The ability of AS to bind nucleolin was suggested to contribute to the tumor growth inhibitory activity on 3LL and A549 human lung adenocarcinoma by altering signal transduction, thereby driving cells into stress condition. AS can affect the nucleolin functions as a cell-surface receptor for a variety of growth factors/chemokines and as a shuttle protein between the cytoplasm and the nucleus by inducing its degradation.

### 4.2. Bacterial HS and Semisynthetic Heparin-Like GAGs

The discovery of a biosynthetic natural polysaccharide produced by some strains of *Escherichia coli* provided a great opportunity to produce “biotechnological” heparin/HS endowed with different biological properties, and to finely dissect structural determinants responsible for anticoagulant/antithrombotic, anticancer and anti-viral activities [214,220,221]. The capsular polysaccharide *E. coli* K5 formed by the repetition of a disaccharide composed of d-GlcA α 1,4-linked to d-GlcNAc, displays the same structure of the natural biosynthetic precursor of heparin, N-acetyl heparosan. Being not decorated with sulfate groups nor epimerized at d-GlcA residues, K5 is biologically inactive with regards to effects on coagulation, proliferation, and inflammation. Actually, this bacterial polysaccharide represents an interesting starting material to produce, via chemical enzymatic technology, non-animal derived-heparin/HS-like GAGs with or without anticoagulant activity [214,221,222,223]. On the other hand, Chinese hamster ovary cells producing natural HS can be metabolically engineered to obtain high-value “designer heparins” endowed with peculiar structural and functional properties [223].

Poggi et al. [224] evaluated the effect of a series of semisynthetic sulfamino heparosan compounds (SAHSs) obtained via chemical modifications of the K5 polysaccharide and characterized by various degrees of sulfation, distribution of sulfates, and molecular size, in lung experimental metastasis with mouse B16-BL6 melanoma cells. Among the compounds tested, SAHS-2 and SAHS-4 displayed remarkable antimetastatic activity related to the degree and ratio of 2-O- and 3-O-sulfation at GlcA residues, although they still possessed some anticoagulant and antiXa activity. However, an LMW fraction of SAHS-4, affinity-depleted of AT-binding sites, conserved antimetastatic activity similar to that of the parent compound, confirming the involvement of other mechanisms not related to the interference with the clotting system in the inhibition of melanoma cell lung colonization.

A number of heparosan derivatives have been reported to have FGF2 antagonistic activity, producing angiostatic effects. For instance, highly sulfated species of the K5 polysaccharide were shown to bind FGF2 and inhibit FGF2-induced endothelial cell proliferation and angiogenesis, likely by interfering with the formation of FGF2/FGFR/HS complexes [220,221,225]. Noteworthy, although various species of N-acetylated, O-sulfated K5 polysaccharides bind FGF1, FGF2, and FGF8b [226], their impact on growth factor-triggered signaling and biological effects may be different, depending on the cellular context, the receptor type, and cell-surface density along with the HS fine structure. Since FGFRs differ in their interaction with HS [227], receptor activation and signaling might consequently vary in their susceptibility to inhibition by HS mimetics. Indeed, Borgenstrom et al. [226] demonstrated that the FGF antagonistic activity of O-sulfated K5 polysaccharides was influenced by the FGF/FGFR species and the cellular context. In particular, FGF8b-induced proliferation of S115 mammary carcinoma cells was markedly inhibited only by the highly O-sulfated K5-OS, which binds FGF8b with high affinity preventing receptor activation. An LMW derivative, obtained via nitrous acid depolymerization of the K5N,OS compound, maintained antiangiogenic activities abrogating the formation of the HSPG/FGF2/FGFR ternary complex with negligible anticoagulant activity. Subsequently, among chemically O-sulfated K5 polysaccharides with low anticoagulant activity, the high-molecular-weight derivative (OS-HMW) was reported to significantly inhibit the metastatic dissemination of human MDA-MB-231 breast cancer cells to the bone, and of mouse B16-BL6 melanoma cells to the lung [228]. OS-HMW antimetastatic effects have been related to heparanase inhibition and to the ability to lower cancer cell adhesion to endothelial cells, and to intercellular adhesion molecule 1 and P-selectin. The K5-NS,OS derivative was shown to inhibit osteolytic bone destruction and tumor growth of the highly bone metastatic model of breast cancer MDA-MB-231(SA) [229]. This effect was associated with the ability of K5-NS,OS to inhibit TGF-β-induced IL-11 production, likely by affecting TGF-β–HSPG interaction by virtue of its high sulfation degree at C-6.

Other studies exploited the versatile K5 polysaccharide to decipher the multi-faced substrate specificity of heparanase [230,231,232]. Using K5 derived substrates, Peterson et al. [231] demonstrated that heparanase cleaves the linkage –GlcA–GlcNS3S (or –GlcNS6S) and the linkage GlcA2S–GlcNS. Moreover, the repeating disaccharide unit IdoA2S–GlcNS was shown to have enzyme inhibitory properties. Of note, as polysaccharides carrying the IdoA2S–GlcNS repeating sequence have no anticoagulant activity or cell proliferation stimulating effect, these studies provided useful information for designing novel heparanase inhibitors devoid of side effects [233].

Studies on the anticancer/antimetastatic activity of variably sulfated K5 polysaccharides also provide information about the signaling and the biological function of their multiple targets. For instance, K5-NS,OS was shown to inhibit the SDF-1/CXCL12 chemokine-mediated B16 melanoma cell proliferation and adhesion to endothelial cells and activated platelets, likely by interfering with the CXCL12–CXCR4 interaction [234]. Consistently, CXCL12 was reported to bind sulfated domains of GAGs with high affinity predominantly through 2-O- and N-sulfate groups [235]. These studies also suggested immunomodulatory and antiflammatory activities of sulfated K5 polysaccharides. Teng et al. [236] described the ability of K5-OS_2_ to stimulate TNF-α and IL-1β production by RAW 264.7 mouse macrophages and to enhance both murine T- and B-cell proliferation. However, a possible contribution of immunostimulating effects on the antitumor activity of these derivatives remains to be defined.

Heparosan derivatives were also used to determine how the sulfation pattern can influence receptor-mediated cell internalization and nuclear localization of heparin-like polysaccharides and consequent biological effects [237]. These findings provide information for designing heparin–drug conjugates as drug delivery vehicles which can also be used in the oncological context.

### 4.3. Synthetic Oligosaccharidic HS Mimetics

Progresses in synthetic chemistry enabled the production of structurally defined HS oligosaccharides, thereby extending investigational opportunities to counteract pathological HSPG functions. Cole et al. [238] synthesized a series of HS oligosaccharides ranging from 7–12 saccharide residues containing a repeating disaccharide unit consisting of l-iduronate 2-O-sulfate linked to d-GlcN with or without N-sulfation. They demonstrated that the inhibition of endothelial cell functions essential for angiogenesis is dependent on specific structural features of the HS fragments. The 12-mer 2SNS emerged in this series as the most potent oligosaccharide in inhibiting endothelial cell migration and signaling induced by key proangiogenic factors such as FGF2 and VEGF165.

Further studies demonstrated the relevance of site-specific sulfation in determining binding preferences and biological effects of synthetic, mono 6-O-sulfated HS mimetic polysaccharides. For instance, the addition of a single 6-O-sulfate moiety at the non-reducing end of an otherwise uniformly 2-O- and N-sulfated dodecasaccharide switched the inhibitory selectivity from CXCL12 to CXCL8, and increased the potency in targeting FGF2-mediated biological activities in endothelial cells, while minimally improving the effects against VEGF165-induced cellular functions [239,240].

Kuhnast et al. [241] reported the in vitro antiangiogenic properties of a rationally designed octasaccharide-based HS mimetic and of its fluoropyridinilated radioactive derivative. The parental oligosaccharide showed strong affinity for VEGF-A, FGF2, PDGF-β, and SDF-1α, as well as potent heparanase inhibitory activity. Both agents similarly inhibited FGF2-induced proliferation of normal human dermal fibroblasts (NHDF). Octasaccharide-^18^F labeling provided useful insights concerning the pharmacodistribution of the HS mimetic. In male Winstar rats, the radiolabeled derivative displayed a long-lasting residence time in the vascular system and progressive accumulation in elimination organs (liver, kidneys and bladder).

Roy et al. [242], in a structure–activity relationship (SAR) study, evaluated the affinity for 22 heparin binding proteins and the heparanase inhibitory activity of three synthetic heparin mimetic hexasaccharides in vitro. The different hexasaccharides contained the same (l-iduronic acid–d-glucosamine)_3_ backbone but varying substitution patterns. Among them, the irregular N-differentiated α pentyl hexasaccharide-3 presenting N-acetylated and N-sulfated d-glucosamines and one non sulfated uronic acid unit, displayed a unique and distinct molecular recognition profile. With respect to the other hexasaccharides, it exhibited the highest affinity for various immunomodulatory/inflammation proteins including bone morphogenetic protein 6 (BMP6), C–C motif chemokine ligand 2 (CCL2), CXCL12, CXCL4, IFNγ, IL-23, P-selectin, VEGF, and Wnt-3a. The atypical conformational orientation of hexasaccharide 3 associated with the two N-acetyl glucosamines at the terminal end, and the internal iduronic acid N-sulfated glucosamine disaccharide, can contribute to its interesting binding profile. In addition, hexasaccharide 3 reduced heparanase activity, exhibiting a noteworthy atypical bell-shaped inhibition curve, which might reflect a different mechanism of action or substrate competition at high concentration.

**Figure 7:**
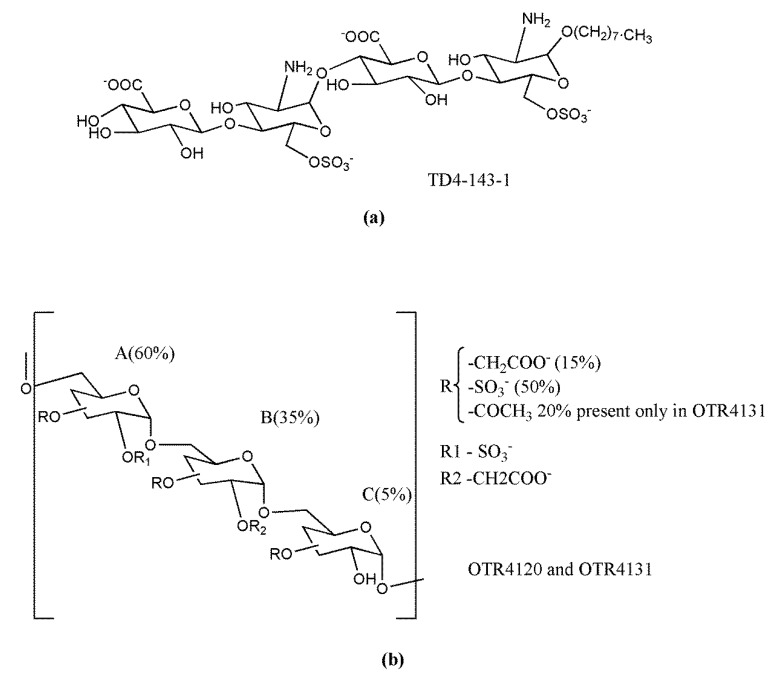
Chemical structure of the synthetic heparan sulfate mimetic TD4-143-1 (**a**). Schematic representation of the synthetic heparan sulfate mimetics OTR4120 and OTR4131 (**b**).

Although rare, naturally occurring N-unsubstituted GlcNH_3_^+^ residues display relevant biological functions. Nadanaka et al. [243] showed that HS structures possessing N-unsubstituted GlcN accumulate in highly invasive human breast cancer cells and evade heparanase-mediated degradation. Indeed, chemically synthetic HS tetrasaccharides containing unsubstituted GlcN residues, GlcAβ1-4GlcNH_3_^+^ (6-*O*-sulfate)α1-4GlcAβ1-4GlcNH_3_^+^ (6-O-sulfate)(TD4-143-1) (Figure 7a), accumulated in lysosomes, inhibited heparanase activity, and suppressed invasion of breast cancer cells in vitro [243]. Noteworthy, since TD4-143-1 tetrasaccharides do not possess the critical structure for complex formation with AT, they are supposed to exhibit no anticoagulant activity.

The synthetic polymers OTR4120 or OTR4131 (Figure 7b) were shown to directly bind the proinflammatory basic chemokine CCL5 (regulated on activation, normal T cell expressed and secreted; RANTES) and to inhibit the the CC-chemokine-stimulated migration and invasion of the human hepatoma cell line HuH7 [244]. These synthetic derivatives of dextran T40, composed of ~200 d-glucopyranose units linked by α-1,6 bonds, are equally characterized by the presence of carboxylate and sulfated groups in extents similar to those found in heparin. The presence of acetate groups increased the hydrophobicity of the OTR4131 derivative without modifying its inhibitory effects on RANTES/CCL5-mediated signaling.

Sheng et al. [245], using a core disaccharide synthetic precursor, produced a novel class of HS/heparin glycomimetics characterized by a highly tunable structure, controllable sizes, and defined sulfation motifs. Among these glycomers devoid of anticoagulant activity, a specific trisulfated HS mimetic was able to antagonize the chemotactic activity of RANTES/CCL5 with a potency similar to that displayed by heparin. This study provided a general strategy for modulating chemokine activity and dissecting the pleiotropic functions of HS/heparin through the presentation of defined sulfation motifs within polymeric scaffolds. Overall, theses studies demonstrated that controlling the positioning and distribution of sulfate groups within the glycopolymers enables the dissection of the anti-inflammatory functions of HS/heparin from the anticoagulant activity.

Other studies, using minimal-sized oligosaccharides of variable but well-defined sulfation patterns, contributed to refining structural requirements for GAG–HGF interactions and to envisaging structural determinants for the rational design of carbohydrate- and non-carbohydrate-based inhibitors of HGF-triggered signaling [246,247].

The synthetic sulfated pentasaccharide EP80061, mimicking natural HS, incorporates the trisaccharidic minimal sequence for heparanase recognition and a 2-deoxy-1*N*-imino glucuronic acid at the reducing end, and displays relevant heparanase inhibitory activity. It demonstrated significant antitumor activity in human A673 rhabdomyosarcoma xenografts, and antimetastatic activity in rat MAT13762 mammary adenocarcinoma and in mouse B16-F10 melanoma models [248].

## 5. Conclusions

Since the discovery of heparin 100 years ago, polypharmacology beyond anticoagulation of this life-saving agent and related drugs was increasingly recognized and characterized. The definition of structural/functional determinants in the heparin and HS molecules, as well as the consequent production of derivatives with reduced or absent anticoagulant activity, prompted an intense chemical synthesis effort, still ongoing, aimed at the identification of heparin/HS mimetics effective in several diseases. Preclinical oncology studies showed that HS mimetics can potentially counteract all steps of cancer development and progression, from local tumor growth, to angiogenesis and invasion, to distant metastatic dissemination. Immunomodulatory properties of these agents can also provide a relevant contribution to their antitumor effects. Several HS mimetics were found to exert antitumor action; however, the most significant effects were reported for their use in combination treatments. These findings support the notion that interfence on the multiple effects of heparanase/HSPGs on tumor cells and the tumor microenvironment represents a valuable strategy to potentiate the efficacy of standard therapies. Notably, heparanase 2, encoded by an HPSE gene homolog, emerged as an additional player in this complex landscape [249]. It does not exhibit enzymatic activity, but displays higher affinity toward HS than heparanase, competing for HS binding, and thus, inhibiting the enzyme activity. Although preclinical and clinical data suggest a tumor suppressor role for heparanase 2, its functional role and effects of its interaction with HS mimetics in vivo remain to be fully elucidated.

Reports of early trials of the first HS mimetics that progressed to clinical evaluation mostly provided good tolerability and hints of anti-tumor activity. Although conclusions about the clinical relevance of HS mimetics in cancer therapy cannot be drawn at present, preclinical and clinical data suggest a few considerations. HS mimetics represent an innovative promising approach still poorly explored in the scenario of cancer therapy. While the first compounds are facing the clinic, much attention should be paid to fully exploit their potential. The antitumor action of HS mimetics appears to be context-dependent, likely specific for certain tumors, and possibly restricted to subgroups of patients in a way reminiscent of the action of molecularly targeted agents. As for the latter therapeutics, a major challenge ahead will be to identify molecular determinants (e.g., the heparanase/syndecan-1 axis in multiple myeloma) associated with a peculiar sensitivity to treatment, and to discover predictive biomarkers. This is of upmost importance to identify appropriate malignancies and to rationally select patient population for clinical trials. HS mimetics are characterized by good safety and tolerability profiles, a feature that renders them highly suitable for inclusion in combination regimens designed to enhance antitumor efficacy of conventional treatments. Moreover, such a feature, together with the unique pleiotropic mechanism of action, is of particular interest in the perspective of precision medicine, where acquired drug resistance and intratumoral heterogeneity are still important challenges. Implementation of preclinical studies applying novel approaches such as patient-derived in vitro and in vivo tumor models is expected to give impulse to HS mimetic research, providing the opportunity to test drug combinations in predictive models that recapitulate key features of human tumors. The peculiar structures of HS mimetics derived from non-animal sources, as well as those of novel synthetic molecules, can potentially reveal context-specific protein interactions, mechanisms of action, and biological effects. The development of orally active non-anticoagulant heparin/HS derivatives represents an additional extremely interesting frontier in the oncologic field in view of long-term treatments and enhanced patient compliance.

## Figures and Tables

**Table 1:** Heparan sulfate mimetics entered into clinical trials

Drug	Company	Phase	Tumors	Half-Life	Administration Route and Dose	Ref.
CX-01 (ODSH)	Cantex Pharmaceuticals	II ^a^	Acute myeloid leukemia	~3 h	i.v.4 mg/kg	[139,186]
Roneparstat (SST0001)	Leadiant Biosciences	I	Multiple myeloma	14–20 h	s.c.25–400 mg/day	[187]
Necuparanib (M402)	Momenta Pharmaceuticals	I/II ^b^	Pancreatic cancer	N/A ^c^	s.c.0.5–5 mg/kg/day	[188]
Muparfostat (PI-88)	Progen Pharmaceuticals Medigen Biotechnology	III	Hepatocellular carcinoma	~8 h	s.c.160 mg/day	[189,190]
Pixatimod (PG545)	Progen Pharmaceuticals	I	Solid tumors	141 h	i.v.25–150 mgonce weekly	[191]

^a^ in combination with idarubicin and cytarabine (NCT02873338); ^b^ in combination with nab-paclitaxel and gemcitabine; ^c^ not available. i.v.—intravenous; s.c.—subcutaneous.
